# Summary of the best evidence for adult nasal high-flow oxygen therapy nebulization management

**DOI:** 10.3389/fmed.2026.1766114

**Published:** 2026-05-20

**Authors:** Sha Xie, Fengmei Li, Zhi Zeng, Dan Wen, Xiaoli Du

**Affiliations:** Intensive Care Unit, Mianyang Central Hospital, School of Medicine, University of Electronic Science and Technology of China, Mianyang, Sichuan, China

**Keywords:** adults, evidence summary, inhalation, nasal high-flow oxygen therapy, nebulization

## Abstract

**Objective:**

To systematically search, assess, and compile the best evidence on the management of nebulization in adult patients on nasal high-flow oxygen therapy, thereby providing a reference for evidence-based nursing practice.

**Methods:**

This evidence summary was conducted following the Joanna Briggs Institute (JBI) methodology for evidence synthesis and was guided by the ‘6S’ evidence resource model. A systematic search was performed in multiple databases, including PubMed, Embase, the Cochrane Library, Web of Science, CINAHL, China National Knowledge Infrastructure (CNKI), Wanfang Data, VIP Database, and the China Biomedical Literature Database, for publications up to September 2024. The study selection process was reported in accordance with the PRISMA-ScR guidelines. Three researchers independently performed the literature appraisal, evidence extraction, and grading of recommendations.

**Results:**

In total, 839 records were retrieved, and 13 articles were finally included, comprising 1 clinical decision, 2 guidelines, 1 team standard, 7 expert consensuses, and 2 evidence summaries. Quality appraisal using AGREE II showed all three guidelines/standards scored above 60% across all domains. Among the seven expert consensuses, most items were rated positively. From the included literature, 26 best evidence statements were extracted and summarized across seven key areas: preparation for nebulization, medication management, selection and setup of nebulization devices, proper usage, monitoring and nursing, management of nebulization in respiratory infectious diseases, and patient education. The evidence levels ranged from Level 1 to Level 5, and the grades of recommendation included both A (strong) and B (weak).

**Conclusion:**

The research has compiled and consolidated the optimal evidence for managing nebulization in adult patients receiving nasal high-flow oxygen therapy. This summary provides a structured compilation of current best practices, primarily derived from guidelines and expert consensus, which can serve as a practical reference to guide the nebulization process for these patients.

## Introduction

High-flow Nasal Cannula (HFNC), a novel non-invasive oxygen therapy technique, delivers a warm, humidified high-flow gas mixture. It is extensively utilized in the management of patients with acute and chronic respiratory failure, as well as those suffering from hypoxemia (1–3). Studies indicate that HFNC outperforms traditional oxygen therapy in enhancing oxygenation and reducing the need for tracheal intubation (4, 5). Recent clinical studies have demonstrated its effectiveness compared to conventional oxygen therapy in specific populations, such as patients with pulmonary embolism and acute hypoxemic respiratory failure (6). In the course of HFNC therapy, the effective administration of aerosolized drugs has become a significant concern and has received considerable attention. Studies have shown (7) that the flow rate configuration of HFNC, the positioning of the nebulizer, and the technique employed for nebulization can significantly influence the outcomes of nebulization therapy. A randomized controlled study demonstrated that the optimal inhalation dose effect is achieved when the gas flow is adjusted to 50% of the patient’s inspiratory flow rate (8). A consensus declaration (9) has been released internationally, providing explicit norms for administering aerosolized medications in multiple respiratory support scenarios. A worldwide survey has indicated (10) that there exists a discrepancy between the clinical application of HFNC aerosol therapy and the current body of evidence, underscoring the critical need for clinical direction regarding aerosol treatments in conjunction with HFNC. Based on a review of the global literature concerning nebulization in adult patients undergoing nasal high-flow oxygen therapy, this research compiles and examines pertinent evidence, aiming to offer guidance for the nursing care of nebulization in these patients.

## Method

### Research purpose

Based on the PIPOST framework, this research constructs an evidence-based inquiry (11). Study Population (P): Individuals undergoing nasal high-flow oxygen therapy with a minimum age of 18 years. Intervention (I): Inhalation therapy with nebulization. Professionals Implementing the Evidence (P): Nursing personnel in units where nasal high-flow oxygen therapy is administered. Outcome (O): Variations in the patient’s sputum index, including aspects such as sputum consistency and the total volume of expectoration. Blood gas index changes (oxygenation index, carbon dioxide partial pressure); inflammatory index changes (procalcitonin, white blood cells, and C-reactive protein); medical staff compliance with aerosol inhalation. The setting of evidence application (S): Departments related to high-flow nasal oxygen therapy use. Type of evidence (T): Guidelines, expert consensus, systematic review, Meta-analysis, evidence summary, clinical decision-making. This study was reported in accordance with the PRISMA-ScR guidelines to ensure methodological rigor and transparency. The protocol for this best evidence summary was registered in the Center for Evidence-Based Nursing of Fudan University (Registration number: ES20258911).

### Search strategy

This evidence summary was conducted according to the ‘6S’ evidence resource model to systematically identify the highest level of available evidence. The search process followed the top-down principle of this model. We first searched for pre-appraised, synthesized evidence from systems such as UpToDate and BMJ Best Practice. Next, we searched for synopses of syntheses and syntheses themselves (systematic reviews, meta-analyses, evidence summaries) from databases like the JBI EBP Database and the Cochrane Library. Subsequently, we searched for synopses of single studies and single studies (original research) from major bibliographic databases. Using “nasal high-flow oxygen therapy/nebulization” and “HFNC/nebulization” as keywords, we searched guideline websites and professional association websites, including the Chinese Medical Guide Website, BMJ Best Practice, UpToDate, the Joanna Briggs Institute (JBI) Evidence-based Health Care Center Database in Australia, the Guidelines International Network (GIN), the Australian Clinical Practice Guidelines (ACPG) Network, the Scottish Intercollegiate Guidelines Network (SIGN), the New Zealand Guidelines Group (NZGG) website, and the National Health Service (NHS) website in the UK. Using a combination of subject terms and free terms, the search terms were “High flow nasal cannula, Humidified High-flow nasal cannula, Humidified High-flow oxygen therapy, High flow nasal oxygen, High flow nasal oxygen therapy, Heated humidified High flow nasal oxygen, HFNC, HFOT, NHF, High-flow nasal oxygen-delivery system” and “nebulizers and vaporizers, vaporizers and nebulizers, vaporizer, inhaler, inhalator, nebulizer, atomizer, inhalation device.” The search was conducted on PubMed, Cochrane Library, Embase, Web of Science, CINAHL, China National Knowledge Infrastructure (CNKI), Wanfang Data, Weipu Data, and the China Biomedical Literature Database. The detailed, full electronic search strategies for all databases are provided in Supplementary File 1. Taking PubMed as an example, the search formula was: (“humidified high flow nasal cannula”[Title/Abstract] OR “humidified high flow oxygen therapy”[Title/Abstract] OR “high flow nasal oxygen”[Title/Abstract] OR “high flow nasal oxygen therapy”[Title/Abstract] OR “Heated humidified High flow nasal oxygen”[Title/Abstract] OR “HFNC”[Title/Abstract] OR “HFOT”[Title/Abstract] OR “NHF”[Title/Abstract] OR “High flow nasal cannula”[MeSH Terms]) AND (“vaporizers and nebulizers”[Title/Abstract] OR “vaporizer*”[Title/Abstract] OR “inhaler*”[Title/Abstract] OR “inhalator*”[Title/Abstract] OR “nebulizer*”[Title/Abstract] OR “atomizer*”[Title/Abstract] OR “inhalation device*”[Title/Abstract] OR “nebulizers and vaporizers”[MeSH Terms]). The search time limit was from the establishment of the database to September 2024.

### Literature inclusion and exclusion criteria

The inclusion criteria were as follows: (i) Patients with nasal high-flow oxygen therapy, aged 18 years or older; (ii) Access to complete literature; (iii) Latest version of guidelines; (iv) Chinese and English literature; (v) Literature types include guidelines, expert consensus, standards, systematic reviews, Meta-analyses, evidence summaries, clinical practice.

The exclusion criteria were as follows: (i) Literature directly translated from foreign languages or repeatedly included; (ii) Case reports, interpretations of clinical guidelines or consensus, abstract versions; (iii) Non-Chinese and non-English literature.

### Literature quality evaluation criteria

(i) Guidelines and team standards were evaluated for quality using the Clinical Guideline Research and Evaluation System II (AGREE II). (ii) Expert consensus literature was evaluated using the six criteria for evaluating the authenticity of opinion and consensus literature from the Joanna Briggs Institute (JBI) Evidence-based Health Care Center in Australia. (iii) Clinical decisions from authoritative databases such as UpToDate and JBI were directly included as high-quality evidence. (iv) Evidence summaries were evaluated for quality using the Critical Appraisal of Summary Evidence (CASE) form.

### The literature quality evaluation process

During the literature quality assessment process, two nursing master’s students who had received training in evidence-based medicine independently evaluated the included literature. After the evaluation was completed, the two parties cross-verified each other’s work in cases where there was disagreement in the assessment opinions, a third reviewer was involved to make a final determination.

### Criteria for determining evidence level and recommendation level

The first and second authors read each of the finally included documents and used the Joanna Briggs Institute (JBI) Evidence Classification and Recommendation Level System (2014) (12) to classify the included original documents into levels 1 to 5, with level 1a being the highest and level 5c being the lowest. If necessary, the original documents would be traced back to determine the level of evidence. The Grade of Recommendation for each summarized evidence statement was subsequently determined. This was based primarily on the corresponding level and quality of evidence, and finalized through research team consensus considering applicability and feasibility. The JBI Grades of Recommendation framework was followed: Grade A: Strong Recommendation – Supported by higher-quality evidence; strongly recommended for practice. Grade B: Weak Recommendation – Supported by evidence, but application may depend on clinical context or resources.

## Results

### General characteristics of the included literature

**Figure 1:**
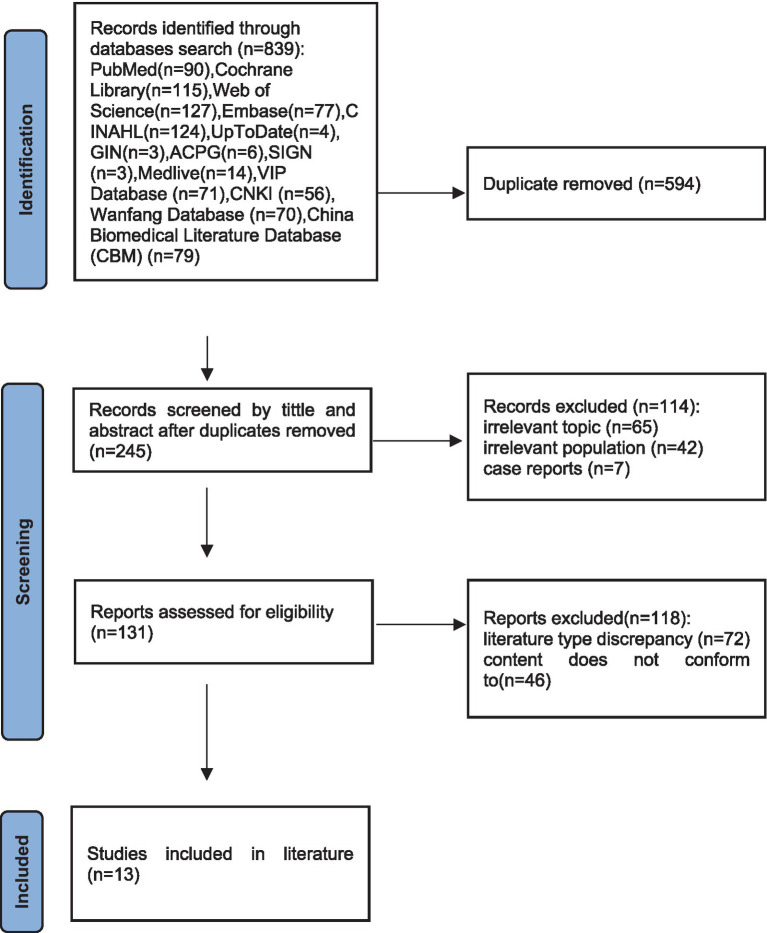
Flow chart of literature screening.

A total of 839 documents were retrieved and imported into EndNote software. After removing duplicate documents and reading titles and abstracts, 245 documents remained. After evaluating the quality of the full texts, 13 documents were finally included. Among them, there was one clinical decision (13), two guidelines (14, 15), seven expert consensuses (7, 9, 16–20), two evidence summaries (21, 22), and 1 team standard (23). The document screening process is shown in Figure 1, and the general information of the included documents is presented in Table 1.

**Table 1:** Characteristics of included articles (*n* = 13).

Included literature	Year	Country	Type of evidence	Literature sources	The topic of the literature	Study design	Key recommendations on HFNC nebulization
Dean et al. (13)	2023	United States	Clinical Decision-making	UpToDate	Adult inhalation drugs	Evidence-based clinical topic review	Use VMN at humidifier inlet; adjust flow to ~50% of inspiratory flow
Oczkowski et al. (14)	2022	Canada	Guideline	ERS	High-flow nasal cannula in acute respiratory failure	International clinical practice guideline	Monitor oxygenation
Katiyar et al. (15)	2022	India	Guideline	Pubmed	Aerosol Therapy Guidelines	National clinical practice guideline	Use VMN at humidifier inlet; do not turn off heater/humidifier; for infections, use mask over circuit during nebulization
Hess et al. (21)	2015	United States	Evidence summary	Pubmed	High-Flow Aerosol Therapy	Narrative review	Place VMN at humidifier inlet; do not turn off heater/humidifier or use Heliox
Qu Xi et al. (22)	2021	China	Evidence summary	CNKI	Aerosol Inhalation Nursing Practice	Evidence summary with JBI methodology	Fast 1 h pre-treatment; semi-recumbent position; monitor vitals; use disposable nebulizers; provide patient education
Li et al. (9)	2023	United States	Expert consensus	Pubmed	Aerosol Drug Delivery Strategies	International multidisciplinary consensus	Use VMN at humidifier inlet or pMDI with spacer; do not interrupt HFNC; set flow to ~50% of inspiratory flow; guide breathing technique
Harb et al. (7)	2020	Egypt	Expert consensus	Embase	Efficacy of Nasal High-Flow Aerosol Therapy	Expert consensus	Use VMN at humidifier inlet; do not interrupt HFNC or turn off heater/humidifier; reduce gas flow to optimize delivery
Mitchell et al. (16)	2014	United States	Expert consensus	Pubmed	Aerosol Therapy	Expert consensus	Perform airway clearance pre-treatment; do not interrupt HFNC for mask nebulization
Chinese Medical Association Respiratory Disease Society (17)	2016	China	Expert consensus	CNKI	Application of Aerosol Inhalation Therapy	National expert consensus	Understand drug incompatibility; prefer single-dose drugs; practice hand hygiene; use disposable nebulizers
Ni Zhong et al. (18)	2020	China	Expert consensus	CNKI	Aerosol Inhalation Therapy for Patients with Novel Coronavirus Pneumonia	Expert consensus	Fast 1 h pre-treatment; semi-recumbent position; use disposable nebulizers
Chinese Medical Association Clinical Pharmacy Society (19)	2024	China	Expert consensus	CNKI	Rational Drug Use in Aerosol Inhalation Therapy	National expert consensus	Understand drug incompatibility and stability; minimize drug mixing
Chinese Medical Association Respiratory Disease Society (20)	2021	China	Expert consensus	CNKI	Clinical Application of Aerosol Expectorant Therapy	National expert consensus	Use disposable nebulizers
Qi Xiao Jiu et al. (23)	2023	China	Team standards	CNKI	Nasal High-Flow Nursing Standards	Organization standard	Provides standardized nursing practice context for HFNC

ERS, The European Respiratory Society; CNKI, China National Knowledge Infrastructure; JBI, Joanna Briggs Institute; VMN, Vibrating Mesh Nebulizer; pMDI, Pressurized Metered-Dose Inhaler; HFNC, High-Flow Nasal Cannula.

### Quality evaluation results of the included literature

#### Quality evaluation results of the guidelines, team standards

Included were two guidelines (14, 15) and 1 team standard (23) for quality assessment, with all three documents scoring over 60% in standardized percentages across six domains. The evaluation results are presented in Table 2.

**Table 2:** Quality assessment results of guidelines and team standards (*n* = 3).

Included literature	Percentage of field standardization %						≥60% field number (*n*)	≥30% field number (*n*)	Recommendation level
	Scopes and objects	Participant	Rigour of the guidelines	Clarity of guidelines	Application of guidelines	Independence of the guide			
Oczkowski et al. (14)	83.33	80.56	83.33	83.33	77.78	100.00	6	6	A
Katiyar et al. (15)	91.67	77.78	94.44	91.67	82.85	83.33	6	6	A
Qi Xiao jiu et al. (23)	80.56	95.83	88.89	91.67	83.33	70.83	6	6	A

#### Quality evaluation results of expert consensuses

Quality assessment was performed on seven expert consensus documents (7, 9, 16–20). Item 6 of Ni Zhong (18) and others were evaluated as ‘No,’ while item 4 of Harb et al. (7) and Li et al. (9) was evaluated as ‘Unclear.’ All other items were evaluated as ‘Yes,’ indicating that the included expert consensus documents have an overall reasonable research design and high overall quality, allowing for inclusion. The evaluation results are presented in Table 3.

**Table 3:** Quality evaluation of expert consensuses (*n* = 7).

Included literature	①	②	③	④	⑤	⑥	Recommendation level
Li et al. (9)	YES	YES	YES	Unclear	YES	YES	B
Harb et al. (7)	YES	YES	YES	Unclear	YES	YES	B
Mitchell et al. (16)	YES	YES	YES	YES	YES	YES	A
Chinese Medical Association Respiratory Disease Society (17)	YES	YES	YES	YES	YES	YES	A
Ni Zhong et al. (18)	YES	YES	YES	YES	YES	NO	B
Chinese Medical Association Clinical Pharmacy Society (19)	YES	YES	YES	YES	YES	YES	A
Respiratory Disease Society (20)	YES	YES	YES	YES	YES	YES	A

① Whether the source of the opinion is clearly labeled; ② Whether the opinion comes from influential experts in the field; ③ Whether the proposed opinion is centered around the interests of the research-related population; ④ Whether the conclusions stated are based on the results of the analysis, and whether the expression of the opinion is logical; ⑤ Whether existing other documents are referenced; ⑥ Whether there are inconsistencies between the proposed opinion and previous literature.

#### Quality evaluation results of clinical decision-making, evidence summary

Included was one clinical decision-making document (13) with high-quality evidence directly included. Two evidence summaries (21, 22)were included, among which the recommended opinions extracted by Hess et al. (21) traced back to 1 evidence summary (13)sourced from UpToDate, directly extracting evidence and evidence levels. Tracing back to another expert consensus (17)from the Chinese Medical Association Respiratory Disease Society, all items were evaluated as ‘Yes.’ One evidence summary (22)was evaluated using CASE, with only the third item evaluated as ‘Not entirely’ and the rest evaluated as ‘Yes.’ Thus, it was included.

### Summary and description of evidence

Through evidence extraction and integration, 26 pieces of best evidence were extracted from seven aspects: preparation before inhalation, medication management, selection and placement of nebulization devices, standard use, monitoring, and nursing, management of aerosol inhalation for respiratory infectious diseases, and patient education. See Table 4 for details.

**Table 4:** Summary of best evidence for management of high-flow nasal oxygen therapy with nebulization in adults.

Evidence items	Evidence content	Level of evidence	Grade of recommendation
Aerosol inhalation preparation	1. It’s advised not to consume food 1 h before undergoing nebulization therapy. Ensure to clean any oral secretions and remaining food particles to avoid vomiting that may be triggered by the air current during nebulization (18, 22).	5b	B
	2. Before the procedure, employ airway clearance methods suitable to the patient’s condition to assist in sputum expulsion (16, 18).	4a	B
	3. For patients without any contraindications, sitting, semi-recumbent or side-lying positions are advisable, ensuring the bed head is elevated at an angle of 30° to 50° (18).	5b	B
Medication management	4. Clearly understand drug incompatibilities and prioritize single-dose medications. (17, 19).	5b	B
	5. When mixing medications in a nebulizer is required, it’s crucial to adhere to the instructions and be knowledgeable about the compatibility and stability of the drugs, aiming to minimize the mixing of different substances (17, 19).	5b	B
Selection and placement of nebulization devices	6. When delivering aerosols via HFNC, the vibrating mesh nebulizer is the preferred choice for HFNC nebulization therapy. (7, 9, 13, 15, 21)	1c	A
	7. When using a pressurized metered-dose inhaler (pMDI) in conjunction with HFNC, it is recommended to use a spacer and place it close to the nasal cannula to ensure that the aerosol flows toward the patient (9).	2b	A
	8. When using a vibrating mesh nebulizer in conjunction with HFNC, it is recommended to place it at the entrance of the humidifier (7, 9, 13, 15, 21).	3b	B
Standard use	9. It is not recommended to discontinue HFNC treatment to use a nebulizer with a mask or nebulization nozzle (7, 9, 13, 15, 16, 18, 22).	2b	A
	10. When nebulizing with HFNC, a mask or mouthpiece nebulization is recommended at the patient’s end unless the patient is intolerant (9).	2c	B
	11. When delivering aerosols via HFNC, it is not recommended to turn off the heater and humidifier (7, 9, 15, 21).	2c	B
	12. It is not recommended to add Heliox (a mixture of helium and oxygen) solely to increase nebulization efficiency (9, 13, 15, 21).	3b	B
	13. To optimize aerosol delivery through HFNC, the gas flow rate can be appropriately reduced (7, 9, 13, 21) so that the set gas flow rate is less than the patient’s inspiratory flow rate (9, 13, 21).	2c	B
	14. Setting the HFNC airflow velocity to about 50% of the patient’s inspiratory flow rate can achieve the optimal inhalation dose (9, 13).	2b	A
	15. When the set gas flow rate is lower than the patient’s inspiratory flow rate, it is recommended for the patient to breathe through an open mouth during HFNC nebulization. Conversely, use an open mouth when the set gas flow rate is higher than the patient’s inspiratory flow rate (9).	2c	B
	16. It is recommended that clinical workers guide patients to take deep and slow breaths during the gas nebulization process, which is beneficial for the deposition of the drug in the lungs (22).	5b	B
Monitoring and nursing	17. During treatment, the patient’s vital signs, such as respiratory rate, oxygen saturation, and heart rate, should be closely monitored to promptly detect and manage any adverse reactions. The treatment plan should be adjusted promptly based on the patient’s condition and current status (16, 22).	5b	B
	18. Dynamically monitor indicators related to hypoxemia, such as SpO2 and the PaO2/FiO2 ratio (14).	5b	B
Respiratory infectious management of aerosol inhalation for respiratory infectious diseases	19. Due to the lack of data, it is difficult to propose recommendations for the combined use of aerosolization and HFNC in these infectious cases, but both have the potential for infectiousness when used alone (21).	3b	B
	20. When performing aerosol therapy, the operator should wash their hands before and after the treatment to reduce the transmission of pathogens between patients (17, 22).	5b	B
	21. When performing HFNC on patients with infectious viral infections, it is recommended that healthcare workers practice personal protection and hygiene during aerosolization (21).	3b	B
	22. For patients with respiratory infectious diseases during HFNC, it is recommended to perform aerosolization separately after stopping HFNC or simultaneously through the HFNC tip covered with a medical mask to prevent the spread of aerosols in the environment. (15, 21).	3a	B
	23. After treatment, the treatment area should be thoroughly cleaned and disinfected to prevent environmental contamination (22).	5b	B
	24. To reduce the risk of cross-infection, it is recommended to use disposable nebulizers (17, 18, 20, 22) (24).	5b	B
Patient education	25. Provide detailed health education to patients, including the correct use of equipment, precautions during treatment, etc., to improve patient compliance (22).	5b	B
	26. Educate patients to follow cough etiquette to reduce the risk of aerosol transmission (24).	5b	B

## Discussion

### Preparations before HFNC nebulization inhalation

Adequate preparation before nebulization is crucial for treatment success. Evidence suggests (16, 18, 22) that measures such as clearing respiratory secretions, oral hygiene, and recommending that patients fast for one hour before treatment are taken to reduce adverse reactions during therapy, such as vomiting. Elevating the head of the bed not only helps avoid tube twisting but also may improve drug delivery. However, there is still a lack of high-quality research evidence on the assessment items before HFNC nebulization inhalation, indicating a need for more research in this field to establish unified assessment standards.

### Use of nebulized medications, selection and installation of nebulization devices

Drug management and the choice of nebulization devices are directly related to the safety and effectiveness of treatment (17, 19). Strict adherence to drug use guidelines, attention to incompatibility, and preference for single-dose drugs are important measures to reduce adverse reactions. Vibrating mesh nebulizers, as the preferred device for HFNC nebulization inhalation (7, 9, 13, 15, 21), have advantages such as no need for external air sources and effective aerosol delivery. However, its high cost may limit its wide application in clinical settings, especially in resource-constrained settings. This creates a gap between the strong evidence-based recommendation and practical feasibility. Furthermore, the recommendation for device placement is technology-specific; placing vibrating mesh nebulizers at the humidifier inlet is optimal, whereas guidance may differ for jet nebulizers. This underscores the importance of matching recommendations to the available equipment. In the future, more high-quality randomized controlled trials are needed to validate the effects of different nebulization devices to guide clinical practice.

### Standard use and monitoring during HFNC nebulization

The HFNC flow rate settings and the patient’s inspiratory flow have significant effects on the effectiveness of nebulization inhalation (7, 9, 13, 21). Aerosol deposition is inversely proportional to gas flow, indicating that caution is needed when setting HFNC flow rates (25, 26). This presents a key clinical dilemma. The flow required for optimal respiratory support may inherently reduce aerosol delivery efficiency. Recommendations to adjust flow to a percentage of the patient’s inspiratory effort (e.g., 50%) aim to balance this but introduce practical complexity in monitoring and implementation. Continuous use of HFNC, employing mask or mouthpiece interfaces, and maintaining the operation of the heater and humidifier are important measures to ensure patient comfort and safety (7, 9, 13, 15, 16, 18, 22). During treatment, nursing staff should closely monitor patients’ symptoms and signs. Treatment plans should be adjusted promptly based on this monitoring to reduce the risk of adverse reactions. Patient-related variables, such as breathing patterns and inspiratory flow, are underexplored but likely significant sources of variability in treatment response, highlighting an area for future research.

### Management and patient education of nebulization inhalation for respiratory tract infectious diseases

For patients with respiratory tract infectious diseases, the management of nebulization inhalation therapy is particularly important. Medical staff should perform personal protection before and after treatment, thoroughly cleaning and disinfecting the treatment area to reduce the risk of cross-infection. Using disposable nebulizers is also an important measure to reduce cross-infection (17, 18, 20, 22, 24). However, the reuse of nebulizers is common in clinical practice, indicating a need for strengthening training on hospital infection prevention and control awareness. In addition, providing detailed health education to patients, including correct equipment use and precautions during treatment, is an important link in improving treatment compliance and effectiveness. The evidence for many of these crucial safety and education items is often graded lower due to its basis in consensus, despite its high face validity and practical importance. This disconnect between the evidence hierarchy and perceived clinical necessity should be acknowledged when applying these recommendations.

### Limitations of the study

This evidence summary has inherent limitations. Firstly, the synthesized evidence primarily stems from expert consensus documents, which represent a lower level in the evidence hierarchy. Secondly, the inclusion was restricted to Chinese and English literature, potentially introducing language bias. As a summary of existing evidence, it does not provide new data on clinical efficacy. Some specific recommendations may face practical implementation challenges depending on local resources. Finally, evidence published after our search date (September 2024) is not included.

## Conclusion

This study summarizes and synthesizes the current best available evidence for the management of adult HFNC nebulization inhalation into 26 key items across seven domains. This evidence, largely drawn from existing clinical guidelines and expert consensus documents, provides a structured reference to inform and standardize nursing practice in this area. However, it is important to acknowledge that a substantial portion of this evidence base originates from expert consensus, which represents a lower level in the evidence hierarchy. Consequently, while this summary offers a valuable consolidation of current expert-recommended practices, the application of these recommendations in clinical settings should be undertaken with careful consideration of local protocols, resource availability, and individual patient factors. The predominance of consensus documents also underscores a significant gap in high-quality primary research. Therefore, the findings of this review not only guide current practice but also clearly highlight the urgent need for more robust clinical studies to strengthen the evidence supporting specific nebulization practices during HFNC therapy.
