# Supplementary material for: Summary of the best evidence for adult nasal high-flow oxygen therapy nebulization management
Source: Front Med (Lausanne). 2026 May 20;13:1766114. doi: 10.3389/fmed.2026.1766114 (PMC13229881; doi:10.3389/fmed.2026.1766114)
Supplement: Supplementary file 1 [file Data_Sheet_1.DOCX]

Search strategy of PUBMED

| **Search strategy** | ("humidified high flow nasal cannula"[Title/Abstract] OR "humidified high flow oxygen therapy"[Title/Abstract] OR "high flow nasal oxygen"[Title/Abstract] OR "high flow nasal oxygen therapy"[Title/Abstract] OR "Heated humidified High flow nasal oxygen"[Title/Abstract] OR "HFNC"[Title/Abstract] OR "HFOT"[Title/Abstract] OR "NHF"[Title/Abstract] OR "High flow nasal cannula"[MeSH Terms]) AND ("vaporizers and nebulizers"[Title/Abstract] OR "vaporizer*"[Title/Abstract] OR "inhaler*"[Title/Abstract] OR "inhalator*"[Title/Abstract] OR "nebulizer*"[Title/Abstract] OR "atomizer*"[Title/Abstract] OR "inhalation device*"[Title/Abstract] OR "nebulizers and vaporizers"[MeSH Terms]). |
| --- | --- |

Search strategy of Embase

| **Search strategy** | 1. exp high flow nasal cannula oxygen therapy/ or (humidified high flow nasal cannula or hfnc or hfot or nhf).ti,ab,kw.  2. exp nebulizer/ or (nebulizer* or vaporizer* or atomizer* or inhaler* or inhalation device*).ti,ab,kw.  3. 1 and 2 |
| --- | --- |

Search strategy of Cochrane Library

| **Search strategy** | #1 MeSH descriptor: [Oxygen Inhalation Therapy] this term only  #2 (humidified high flow nasal cannula or HFNC or HFOT or NHF):ti,ab,kw  #3 #1 or #2  #4 MeSH descriptor: [Nebulizers and Vaporizers] this term only  #5 (nebulizer* or vaporizer* or atomizer* or inhaler*):ti,ab,kw  #6 #4 or #5  #7 #3 and #6 |
| --- | --- |

Search strategy of CINAHL

| **Search strategy** | TS=("humidified high flow nasal cannula" OR "high flow nasal oxygen" OR HFNC OR HFOT OR NHF) AND TS=(nebulizer* OR vaporizer* OR atomizer* OR inhaler* OR "inhalation device") |
| --- | --- |

Search strategy of Web of Science

| **Search strategy** | ( (MH "Oxygen Inhalation Therapy") OR TI ( "humidified high flow nasal cannula" OR HFNC OR HFOT OR NHF ) OR AB ( "humidified high flow nasal cannula" OR HFNC OR HFOT OR NHF ) ) AND ( (MH "Nebulizers and Vaporizers") OR TI ( nebulizer* OR vaporizer* OR atomizer* OR inhaler* ) OR AB ( nebulizer* OR vaporizer* OR atomizer* OR inhaler* ) ) |
| --- | --- |

Search strategy of 中国知网 (China National Knowledge Infrastructure, CNKI)

| **Search strategy** | SU=('经鼻高流量氧疗' + '高流量鼻导管氧疗' + 'HFNC' + '湿化高流量鼻导管氧疗') AND SU=('雾化' + '吸入' + '雾化器' + ' nebulizer') |
| --- | --- |

Search strategy of 万方数据 (Wanfang Data)

| **Search strategy** | 主题:(经鼻高流量氧疗 OR 高流量鼻导管氧疗 OR HFNC OR 湿化高流量鼻导管氧疗) AND 主题:(雾化 OR 吸入治疗 OR 雾化器 OR nebulizer) |
| --- | --- |

Search strategy of 维普数据 (Weipu Data)

| **Search strategy** | M=(经鼻高流量氧疗 OR 高流量鼻导管氧疗 OR HFNC) AND M=(雾化 OR 雾化吸入 OR 雾化器) |
| --- | --- |

Search strategy of 中国生物医学文献数据库 (China Biomedical Literature Database)

| **Search strategy** | #1 “经鼻高流量氧疗”[常用字段:智能] OR “高流量鼻导管氧疗”[常用字段:智能] OR “HFNC”[常用字段:智能]  #2 “雾化”[常用字段:智能] OR “雾化吸入”[常用字段:智能] OR “雾化器”[常用字段:智能]  #3 #1 AND #2 |
| --- | --- |
